# Functional and recreational dog walking practices in the UK

**DOI:** 10.1093/heapro/daaa051

**Published:** 2020-05-03

**Authors:** Carri Westgarth, Robert M Christley, Garry Marvin, Elizabeth Perkins

**Affiliations:** 1 Department of Epidemiology and Population Health, Institute of Infection and Global Health, University of Liverpool, Liverpool CH64 7TE, UK; 2 Institute of Veterinary Science, University of Liverpool, Liverpool CH64 7TE, UK; 3 Department of Life Sciences, University of Roehampton, London SW15 4JD, UK; 4 Department of Health Services Research, Institute of Psychology, Health and Society, University of Liverpool, Liverpool L69 7ZX, UK

**Keywords:** dogs, exercise, health, qualitative research, walking

## Abstract

Dog walking is a popular everyday activity known to contribute considerably to human health through motivating substantial additional physical activity. However, despite recognition that walking with a dog is substantially different from walking without a dog, little is known sociologically about the practices of dog walking. This study used in-depth interviews with 38 dog owners, combined with autoethnographical observation of dog walking. The aim was to investigate the types of dog walks that occur and the implications of this for the promotion of dog walking to increase human and animal wellbeing. Two distinct types of dog walking were found that had differing influencers and resulting experiences. Functional walks were purposed through feelings of guilt to provide the dog with a convenient form of exercise but were less pleasurable for the owner. In contrast, recreational walks provided significant owner stress-relief and were longer, typically during pleasant weather and at weekends, in less urban environments, and involved more members of the household. Limitations on time availability, conducive weather or accessibility of desirable physical environments for dog walking, generated functional rather than recreational dog walks. These findings have implications for interventions aiming to promote dog walking and for policy relating to the availability of safe and suitable green spaces for encouraging dog walking.

## INTRODUCTION

The promotion of exercise is a key component of both the prevention and treatment of illness, yet interventions to encourage walking have rarely shown to sustain the activity in the long term (Ogilvie *et al.*, 2007). Dog walking appeared in the 19th century, as a way of enabling the pet dog to enter public spaces in a more controlled and supervised way (Howell, 2015). Dogs appear to provide a unique vehicle for encouraging owners to undertake regular, sustained and physical activity (Peel *et al.*, 2010). In general, dog owners undertake considerably more walking than people without a dog; however, many owners do not walk with their dog regularly (Christian *et al.*, 2013; Westgarth *et al.*, 2019a). Research is now underway in order to understand how to promote dog walking as a means of enhancing both human and animal wellbeing (Levine *et al.*, 2013; Westgarth *et al.*, 2014; Christian *et al.*, 2018).

As an everyday activity, walking has escaped detailed sociological analysis until recently (Green, 2009; Vergunst and Ingold, 2016). Walking is inherently a social activity, often performed with others, and there is a sense of pleasure gained from sharing the experience (Darker *et al.*, 2007; Vergunst and Ingold, 2016; Grant *et al.*, 2017). Walking with a dog is an example where social relations during walking cross-cut between human and animals, between the owner and their companion (Vergunst and Ingold, 2016). Walking can occur both as an integrative practice and a dispersed practice (Green, 2009; Harries and Rettie, 2016). Rambling would be an example of an integrated practice, where the purpose of the walking is actually to experience the walk. In contrast, dispersed walking is walking that occurs as part of a different integrative practice, such as shopping or travelling to work; walking is not the purpose of the practice but a means to achieve it.

The differences between dispersed and integrated walking practices may have implications in interventions aiming to promote physical exercise. As such, physical activity researchers often draw a distinction between walking performed for ‘recreation’ or ‘leisure-time’ and walking performed more purposefully as a mode of ‘active transport’, ‘travel’ or ‘commuting’ (Giles-Corti and Donovan, 2003; Ogilvie *et al.*, 2007). Walking with a dog has been assumed to be leisure-time or recreational physical activity [e.g. (Cutt *et al.*, 2008a; Cleland *et al.*, 2010; Reeves *et al.*, 2011)]; non-exercise-related walking (Thorpe *et al.*, 2006); chores/errands (Tudor-Locke and Ham, 2008); and commuting/transport physical activity (Corseuil *et al.*, 2011). Thus, there are clearly questions regarding the formats dog walking occurs that requires further examination.

Although a significant proportion of households own dogs [UK 24% (PFMA, 2017), USA 38% (AVMA, 2018) and Australia 39% (AMA, 2019)], there has been little investigation into the specific nature and purpose of dog walking (Westgarth *et al.*, 2014). It is recognized that walking with a dog is different to walking without a dog (Lim and Rhodes, 2016). Our previous research suggests owners walk their dogs due to a strong sense of responsibility engendered by the reciprocal social and emotional relationship with them (Westgarth *et al.*, 2019b). However, not all dog owners walk their dog regularly and previous qualitative studies have identified further barriers and motivators to walking with a dog including the availability of dog-supportive environments [e.g. (Cutt *et al.*, 2008a; Degeling and Rock, 2013; Westgarth *et al.*, 2017)]. However, there has been no investigation of the types of dog walking experiences undertaken when dog walks do occur and how these may be influenced by factors, such as the environment.

This new article examines, through discussions with dog owners about their dog walking practices, what a *dog walk* is and how they walk with their dogs. This article follows from our previous publication that describes how motivations for dog walking are based upon how the individual dog’s needs are constructed, and the importance owners place on walking their dog (in particular the positive outcomes owners gain from it), in the context of their own lives (Westgarth *et al.*, 2017). This new article aims to analyse how the needs of the owner and the dog interact to shape the ‘dog walks’ that result. Second, the article aims to demonstrate how the physical environment interacts with and influences dog walking practices. This information will allow us to understand how dog walking is experienced and the forms it can take, in order to discuss the implications for promoting dog walking as a population health strategy.

## MATERIALS

The methods for the study have been described in more detail (Westgarth *et al.*, 2017, 2019b). Briefly, 38 people were interviewed (mostly by C.W. but some by R.M.C.) about their relationship with their dogs and involvement in dog walking. This included in-depth interviews with multiple people within participating households (usually in their home) who were recruited via social media and leaflets/posters in community areas, and shorter interviews with dog owners approached out walking their dog in city parks or representing their breed at a dog show. Participants owned a variety of dog breeds, and included adult males, females and children, from a range of sociodemographic backgrounds and contexts, and varied in how often they walked their dogs, from never to several times a day. Participants lived mainly in Merseyside and Cheshire but interviews were also conducted with participants from wider across the UK. In the UK, dog walking areas in typical neighbourhoods include streets with pavements/sidewalks and local small grassed parks, sports fields or farmer’s fields. Interspersed between neighbourhoods are larger designated country parks such as beaches or woodland. In addition, there are national parks of hills and moorlands. It is culturally typical for dogs to be walked off-leash unless on streets (although not compulsory) or in a designated on-leash only area. Enclosed ‘dog parks’ are currently very rare.

The individual dog ownership and walking-experience was discussed in detail and, where possible, the first author also accompanied the participants on a typical dog walk. The data collection was supplemented by an autoethnography of the first author’s own dog walking experiences over 2 years recorded in a diary, whilst owning three dogs and with a baby/toddler. The study was approved by the University of Liverpool Veterinary Ethics Committee (Project code VREC121) and written informed consent from participants was obtained, or recorded verbal consent if they were interviewed whilst out walking.

All interviews were audio-recorded and fully transcribed. Autoethnographical diary entries and interview transcripts were used to inductively code emerging themes with the assistance of NVIVO to manage data, using initial line by line coding by the first author and then subsequent analysis by both C.W. and E.P. into higher analytic themes. A grounded-theory approach was used (Charmaz, 2006) in terms of collecting and analysing data concurrently and sampling purposefully according to emerging concepts and missing participant attributes. Triangulation occurred through comparing the interview transcripts with personal dog walking diary entries of the first author and also ethnographical notes made about wider conversations about dog walking.

## RESULTS

Participants collectively described a perception that, as a general rule, a dog needs a walk every day; however, this may be a recent societal belief:

I can’t remember when people started saying, ‘You have to take your dog for a walk.’ […] There wasn’t so much traffic, and they mostly laid around in the garden with the gate open. […] They might have followed their owner to the shops and back, but nobody actually that I can remember particularly made a great point of taking their dog out every day.

(Grace)

**Fig. 1.**
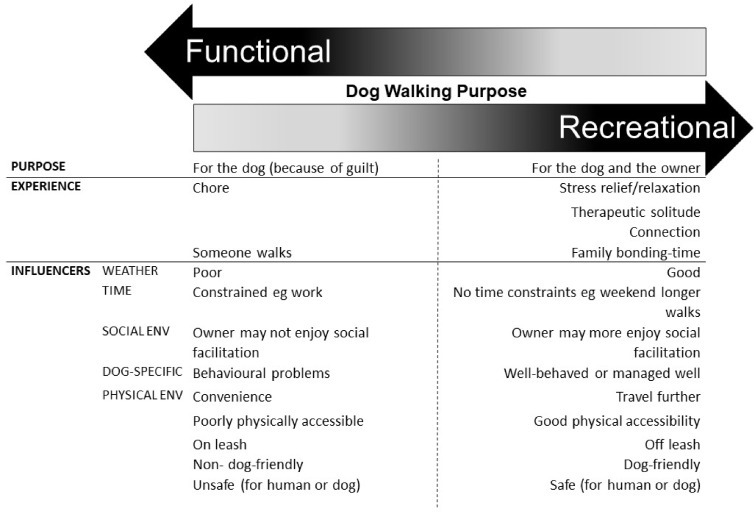
Key themes of functional and recreational dog walking.

Thus, there was an intention to perform a dog walk daily; however, the nature of that dog walk varied depending on the past personal experiences and individual needs of each particular owner (or household of owners) and dog(s). Within this variability, there emerged two key types of walk; functional dog walking and recreational dog walking. Themes were identified that illustrated how functional and recreational dog walks had different purposes, and were experienced differently (see Figure 1). There were also thematic conditions that influenced whether a functional or recreational dog walk occurred. In the simplest form, a functional walk is that primarily performed for the benefit of the dog, under time pressure, in bad weather, in a convenient location. At the other extreme, a recreational walk was that performed for the benefit of both the dog and the owner, without time pressures, in nice weather, and in a pleasing location.

### Purpose

Functional walks were primarily for the purposes of meeting the needs of the dog. In contrast, Recreational walks catered for both the dog owner’s needs as well as those of the dog. They constituted an integrated practice where the purpose of the walk for the owner was to enjoy the walk, not just meet the needs of the dog to have a walk.

But that was a completely different walk than the ones I do in the woods. The ones that I do when I’ve got to go to work or just to the common or just to the fields to me are literally just for Bear. Even though I enjoy it because I’m walking with Bear and I’m out but they were just functional walks. The walks that I did in the woods were for his benefit and for my benefit and they were a completely different feeling for me than the functional ones.

(Mary)

At the most basic level, a functional walk met the physical need for the dogs to urinate and defecate in a place deemed appropriate by the owner. However, the primary purpose of a functional walk was usually to provide physical exercise for the dog. Participants described some activities that might be undertaken during a functional walk to enhance the experience for the dog including playing with a toy, retrieving a ball or running alongside a bike.

They’re the cheap walks; they’re the walks that, as a professional, I hate people doing. You drive or walk to a field, spend twenty minutes throwing the ball, for your dog, and then you’re taking them back home.

[Nadine (dog behaviourist)]

Functional walks were motivated by the desire to avoid feeling guilty were the dog not to be walked.

First day back to work after the break and it was VERY difficult to get up at 5.45am so that I could go walk the dogs in the dark and rain before work, especially as the toddler carried on sleeping for once so the house was all peaceful but I still had to get up on my own. So why did I do it? GUILT. Pure guilt that I could not have left the dogs all day without having been out for a walk.

(Ethnographic diary)

Functional walks were often treated as a chore because they were not as enjoyable to the owners in the same way as recreational walks were, rather perceived as a contractual obligation part of the responsibility between the owner and the dog.

Functional dog walks were also usually taken close to home, in local parks or streets, because these were the most convenient places when short on time:

Interviewer:So is this your local park?

Graham:Yes, this is the one I bring them to when I’ve got less time to spend. When I’ve got more time I’ll take them, obviously, [bigger] Park or I’ll take them out and do different things.

Recreational walks felt different to functional walks and were typically performed in more preferable locations (the influence of the availability of different physical environments will be discussed later). Recreational walks were used to de-stress and relax an owner, in particular after work or at weekends. Dog walks were often described as relaxing, but this was contingent on them not feeling rushed or otherwise hindered or time pressured by the need to undertake other activities, for example get to work:

I love the walk after work—unless the weather is like really horrible and it’s dark in the winter—that after work walk is the best. Like the one before it is just another pressure of another thing to do but after work like you’ve made a break with your day and I love that. It does, it completely clears my day away.

(Diane)

Recreational walks were often termed therapeutic, especially if providing a moment of solitude to think through one’s thoughts:

I do find it therapeutic. Because if you are walking around by yourself, you’ve got your own thoughts for about an hour, or for an hour and 10 minutes or however long it is you can walk round. And you must know that, you’re thinking about a multitude of things, whether it’s past mistakes, things that you’re going through now, you know if … it helps just for that solitude.

(Charles)

Social connections with other people out walking also occur as a result of dog walks (reported in Westgarth *et al.*, 2017), and this occurred during both functional and recreational walking. Recreational walks in particular were also often used as a period of family bonding time when household members walked as a group when time permitted, such as at the weekends. In contrast, since the primary purpose of a functional walk was to meet the dog’s needs rather than the needs of the owner(s), it was common to delegate the activity to one person, ‘as long as someone walks the dog’:

I’ll take Ryan somewhere, and Jake will take the dogs somewhere, and we’ll divide and conquer.

(Nadine)

Adding further complexity, taking this approach to ‘divide and conquer’ may actually enable the dog walker to experience a more recreational dog walk than perhaps would have been available to them otherwise. However, this attitude to dog walking may be a barrier to the full potential of beneficial impacts of dog ownership on household members being attained as it is perceived that they do not all need to participate in the dog walk.

In addition to social contact with other walkers, dog walking brought participants into connection with nature and the environment which was perceived as pleasure-enhancing and contributing to the recreational experience, in particular noting moments that wouldn’t otherwise have been experienced:

I like looking at maybe a shaft of sunlight that comes through the trees and lights up maybe one specific area, or you know… Or the change in the scenes.

(Charles)

In these periods of heightened awareness, participants described how they connected to the present moment during the dog walking experience. However, these were created not just by recognition of the often pleasant surroundings, but also enhanced by their observation of, and connection with, the dogs themselves and pleasure shared vicariously:

And it’s just…you know people don’t remember how to enjoy themselves like that, but dogs always do remember to enjoy themselves and have fun, and play, and enjoy the fresh air.

(Alice)

In summary, recreational walks were described as those from which both the owner and the dog derived significant benefits and were particularly pleasurable. Functional walks were a different type of walk that was perceived as an obligated duty to benefit the dog.

### Influencers

Certain environments and contexts were deemed more conducive to recreational than functional dog walks, and thus could influence whether a walk was experienced as more functional or recreational. When faced with challenges, owners tried to find ways round these so that the walk could be experienced as recreational.

#### Weather

In poor weather walks were often more functional in nature and shorter in duration:

If it’s raining in the morning when he takes them out, he goes out for less time but he takes the flicker.

(Fiona)

At the other end of the spectrum, dog walks were longer during nice weather. Participants could also mitigate the effects of inclement weather by wearing appropriate clothing, adjusting their walk time, or changing location to in order to better manage the consequences of the bad weather and avoid wet or muddy conditions, and thus have a more recreational experience:

I don’t like the wet, so let’s say the forecast today said that it’s going to be raining this afternoon or go colder or whatever, I’d take him out this morning, but if the forecast said, you know, it’s going to be much brighter this afternoon then I would actually take him out this afternoon. I do that because I can enjoy the walk.

(Helen)

#### Time constraints

When time was constrained such as walks before work, dog walks became functional:

On the functional ones […] it would be when I fit it in and when I could do it, so it wouldn’t be a set pattern thing. On the days where I’d have to take him before I’d go to work […] that would be in the morning, yes, when I first got up before going to work, I’d take him to the common and I’d know that he’d be all right then, set for the day.

(Mary)

The more functional perception of these walks was due to the sub-ideal times that these walks often had to be conducted, such as in darkness in winter:

I have a problem. It was very very dark in the woods at 6.30 this morning. I couldn’t see a thing, even with a head torch. It wasn’t very enjoyable, to the point of probably dangerous if I tripped or was attacked by a bad character. In previous years my husband and I walked together, and walked a bit later, as we didn’t have the baby to look after and I didn’t have to be in work by 8am.

(Ethnographic diary)

In contrast, weekend walks were often longer those in the work week, at least for those who worked during the week. Fewer time constraints, such as retirement, meant more flexibility to arrange the timing of the daily walk so that it was more recreational in nature:

#### Dog-related factors

Dog behavioural problems were found to make dog walking less pleasurable for the owner. Dealing with behavioural problems often led to dog walks becoming much more functional in nature, e.g. keeping the dog on a leash and avoiding popular dog walking places where other dogs might be:

[Dog walking] calms me down and I get nice and de-stressed. Originally, with Jack, I would be…always on edge; it felt like a ninja just looking for the nearest escape. The amount of times I ended up being in a bush or up someone’s driveway, to avoid someone else’s dogs, so that one was always stressful.

(Nadine)

Owners had multiple strategies for dealing with behaviour problems of their dogs, such as avoiding trigger contexts, or following specific treatment training methods to improve the situation. To assist with this, they sought out and used a range of aids (collar and lead, head collars, harnesses), in order to make the dog walk more enjoyable and recreational in nature:

She is also now completely deaf I think…cannot even hear the whistle. So she does not come when she is called anymore, which makes the walk less enjoyable if there are people and dogs around that she is happily mooching up to but we need to call her back as we can sense they would prefer not to interact. We now walk her on a harness and mostly keep her on a lead unless nobody else is around.

(Ethnographic diary)

#### Social environment

Facilitated social interaction with household members has been discussed earlier as resulting from the recreational dog walking experience. Social facilitation with other people and dogs is also a recognized outcome of dog walking (Westgarth *et al.*, 2017), but it was also found to influence the perceived functional or recreational nature of the dog walk. Some participants found meeting other people and dogs on walks pleasurable, but others did not:

When you’re out and your expectation is there’s no one else there; 5:00 in the morning or something. You’re out, you’re walking at a nice pace and, all of a sudden, this dog darts out of nowhere, crashes into the back of you, nicks your dog’s toy and pisses off. It’s just like, “Brilliant, peace is shattered.

(Jake)

#### Physical environment

Appropriate physical environments for dog walking were discussed and their availability and accessibility were linked with the functional or recreational nature of walks performed in them; accessible, safe and pleasant spaces may produce more enjoyable recreational walks. However, nearby convenient, but less suitable, dog walking spaces may only facilitate functional walking, or even put owners off taking their dogs for a walk altogether:

Walking around here, it’s horrible, isn’t it? You can’t talk to each other, you’ve got traffic everywhere […] the fields are used by people on dirt bikes and stuff […] I think that’s probably the reason why we don’t go walking; well, we didn’t go walking much. Going out around here was like you’ve got to dodge the people that don’t like dogs; you’ve got to dodge the people that are crooked; you’ve got syringes all over the floor.

(Jake)

Walking on the streets was also viewed as providing only a minimal functional walk because it was perceived as boring for the dog:

Well, I don’t think walking on the pavement is very interesting for them. If I see a dog walking on the pavement I think it must be really boring, being on your lead.

(Fiona)

The more appropriate a space was deemed for dog walking, the more recreational in nature the walks performed there became. For a person who lived close to a great dog walking space, even local convenient walks could be recreational in nature. In particular, participants felt their dogs needed to be off-leash if it were safe to do so. Participants perceived a need for their dogs to have some time without constraint to run and explore:

I look forward to going to the park and just see the dogs going running.

(Child)

A key aspect contributing to a dog walk being perceived as recreational was the owner enjoying seeing a dog running off-leash in safe green environments. Participants were concerned about the way in which anti-dog legislation, such as banning dogs from areas or leash restrictions, might impact negatively on the dog’s wellbeing. Perceived good dog walking locations had interesting scenery and circular routes to avoid repetition. Dog walking locations also had to be safe for the dogs—and ‘dog friendliness’ of walks was felt to be important, mainly encompassing aspects of being safe such as suitable walking surfaces, and avoiding poisons, livestock and vehicles, so that owners could relax and enjoy themselves.

Suitability of a location for dog walking appeared to often trump accessibility of the location:

It’s why I’m happy to get in the car and go to somewhere more remote because it means he’s safer and it’s more fun for me because it’s more fun for him.

(Nina)

However, participants’ fundamental access to dog walking locations varied, for example depending on whether the owner had a car. A 5- to 10-min drive in a car was viewed as feasible for regular walking, but if the owner did not drive or preferred not to use the car, a suitable dog walking location needed to be closer. Many had chosen to live near ‘good’ dog walking locations, suggesting that accessibility of suitable dog walking locations may influence where a dog owner lives if they have an ability to choose.

Locations deemed good for dog walking also needed to be accessible for the owners, e.g. health needs or the needs of walking with children or babies, if a recreational or even a functional walk was to be actualized.

It had to be much more on the streets as I got less able […] It’s very difficult for me to walk on uneven ground.

(Grace)

Equipment for human physical needs such as sticks, walking frames or wheelchairs, buggies and slings were used to try to mitigate accessibility issues:

Yeah this kind of walking it was a lot easier to have him in a carrier or a sling than it was to bring the buggy. We’ve got a big three wheeler thing… it can handle the flat parts of here but not so much the bumpy bits.

(Nadine)

Dog-free children’s play areas were felt to cause conflict if walking with both a dog and a child. There were also concerns raised about the increasing number of specific off-leash dog parks, and the appropriate design and management of these so that it does not encourage dogs to come into conflict with each other.

The desire for improved physical environments for accessible dog walking, including level paths for ease of walking or pushing prams, seats for resting, bins for faeces disposal and parking, were discussed by most dog owners, as these made for a more recreational dog walking experience. Some owners also liked their dog walks to challenge them in some way, or be purposeful beyond that of just walking (e.g. to pick something up from a shop or stop and have a cup of tea, adding a further element of purpose and perhaps relaxation).

In summary, the fundamental distinction between functional and recreational walks was the intended purpose of the walk—primarily for the dog, or for both the dog and the owner. However, this did not mean that owners could not benefit from a functional walk; any dog walk can be beneficial in terms of physical activity and stress relief (see Westgarth *et al.*, 2017); however, particular dog walks under certain conditions were perceived as more strongly eliciting owner enjoyment as well as being good for the dog. Changing circumstances even during a walk could transform it, e.g.: sudden heavy rain so going home early; having your upcoming plans cancelled so that the walk can continue for longer; or coincidentally meeting a friend and walking together. Dog owners responded to perceived challenges and barriers to dog walking, not so much by not walking, but by modifying *how* they walked. These adjustments were used to mitigate challenges and balance recreational/functional practices as desired. Most dog owners were able to find ways to get their dog walked somehow, even if it gave no great pleasure to the dog walker, and became a functional walk. However, this required the space, time and resources to be flexible in how dog walking occur, which is not always available.

## DISCUSSION

It is clear from our study that modern dog walking has continued to evolve since its emergence in the late 19th century (Howell, 2015) and dog walking today is not one process or event. Two types of dog walking practices have been identified for the first time: functional and recreational. At one end of the spectrum dog walks can be performed for a simple functional purpose, usually to meet the perceived practical needs of the dog to undertake some exercise and excretion. Dog owners also talked about a fully integrated dog walking practice in which the walk was lifted out of its everyday nature and transformed. At the end of the spectrum, there are dog walks that are highly recreational and performed for the purpose of leisure, stress relief and joy that are co-created by the dog-owner dyad. Recreational walks go beyond fulfilling the perceived basic needs of the dog, and provide an opportunity for additional beneficial outcomes for the dog owner such as relaxation, stress relief, heightened awareness of the present moment and vicarious pleasure; in short, many mental health benefits. In contrast, functional walks are performed as quickly and efficiently as possible, at a balance point between minimizing effort by the owner and maximizing benefit for the animal.

It is usually the case that an owner mixes functional or recreational dog walking activity with each of the types of walk predominating at different times. For some participants, such as someone who is retired with time to spend and who lives within easy driving distance of an interesting and pleasant dog walking environment, most walks may be largely recreational in nature. For someone who lives next to a large park, the before work walk may be classed as functional and the after work as recreational, despite being conducted in the same space. For an owner who work long hours and does not live within walking distance of a pleasant walking space, all dog walks may be functional except those at weekends that require significant travel to a better location. One walk may even perhaps have functional and recreational elements. The functional and recreational meanings of walking in the sense we have described here emerge from the action of walking with a dog and are bound up with the special kind of walking that is dog walking. More fundamentally, the presence of a dog brings specific components to walking not found elsewhere: it provides a unique functional purpose (to meet the perceived needs of the dog). Similar to other social walking experiences it also produces positive feelings of wellbeing through sharing an experience of intrinsic value.

Previous studies have identified that benefiting from owning a dog (or sharing caring for it) does not depend on walking with it (Degeling and Rock, 2013; Degeling *et al.*, 2016). Further, other work has highlighted the barriers and motivators to walking with a dog (Cutt *et al.*, 2008a; Degeling and Rock, 2013; Westgarth *et al.*, 2017), but have not previously described how these barriers and motivators may influence different dog walking experiences and shape different walk types when dog walks do occur.

Returning to the literature on the sociology of walking, we can compare our findings with other dispersed and integrative walking practices (Green, 2009; Harries and Rettie, 2016). ‘Recreational’ walks as constructed in this research parallels the integrative practices described by (Harries and Rettie, 2016), where the purpose of the walk is to enjoy the walk. In contrast, functional dog walks which aim to meet the needs of the dog fall within the Harries and Rettie’s definition of dispersed walking within the integrative practice of exercising the dog. This fits with the observation that for many of our participants dog walking ‘doesn’t feel like exercise’ (Westgarth *et al.*, 2017) since it is performed with the dog as its focus. In contrast, recreational walking is a more embodied practice where the walk is the goal and the subjective sensation of the movement of the body (or in our case bodies, both human and animal) through that environment, and resultant benefits to the mind an integral part of the experience (Green, 2009). Goode also described playing with his dog as an autotelic activity, an action that has no end other than its own production (Goode, 2007). Functional walks may be a construction of the necessity of work that has to go into maintaining a pet dog in order to at other times gain the valued pleasure and play associated with pet-keeping (Tuan, 1984). In particular, the freedom and play afforded by letting a dog off-leash is a noted point of contention by dog owners throughout the historical emergence of dog walking in the urban areas (Howell, 2015).

The distinction between ‘functional’ and ‘recreational’ dog walks has significance for the design of interventions to promote walking. Given the complexity of dog walking the effects of promoting dog walking as a public health intervention are unclear. The construction of a dog walk as either functional or recreational is a personal experience, unique to each owner, it is not something easily legislated for or promoted on a population level.

Dog walking interventions trialled thus far have tapped into an owner’s desire to exercise their dog and thus may have targeted increasing functional walking. The small gains identified from this may amount to a few extra minutes of physical activity each day (Rhodes *et al.*, 2012). Perhaps the most suitable health promotion strategy here would be using the needs of the dog to leverage functional walking for initiating dog walking in those owners who do not currently walk their dog. It may also be argued that basic functional dog walks are more likely to be maintained as these are seen as essential to the dog, rather than a luxury. Although it is difficult to predict, it is possible that as an owner increases their functional walking frequency they may begin to perceive personal benefits and experience enjoyment and thus progress to undertake more walking, of now a recreational nature. However, it is very difficult to predict the effects, and if functional dog walks are less enjoyable for the owner, it could be argued that recreational walking is the better target for interventions. There is also a danger that a mis-applied focus on increasing dog walking in dog walkers may result in walks once experienced as a recreational activity becoming more functional in experience and less-enjoyable for the owner. In addition, by promoting functional walking, strategies that increase exercise for the dog (playing with a ball) but are not highly beneficial for the owner in terms of exercise (standing in a dog park throwing a ball) may inadvertently be encouraged, as is often observed in dog parks (Evenson *et al.*, 2016).

In contrast, for the greatest benefit to owner and dog wellbeing, theoretically interventions should be directed towards increasing the recreational type of dog walks. Recreational walks are considered the most enjoyable and beneficial for both owner and dog, and our data show these are likely to be longer and may include, and thus benefit, multiple family members. They are also the most enjoyable for owners, so perhaps are most likely to be maintained longer term. However, increasing recreational dog walking may in practice be difficult to achieve, as despite being enjoyable and thus likely to be the most motivating, recreational walks require more time. Owners may also not have suitable locations for recreational walking available to them; accessibility is necessary as distance to suitable walking locations is associated with increased outings (Neuvonen *et al.*, 2007; White *et al.*, 2018). Perhaps the most suitable situation for targeting recreational walking is in current dog walkers who would like to walk even more.

Different stakeholders may have particular roles to play in promoting dog walking. Public health or animal welfare advocates must realize that any strategy aiming to promote dog walking needs to consider exactly what type of dog walking is to be targeted, in the same way that strategies to promote walking (without a dog) for transport, and walking for recreation, would arguably need to be quite different. Veterinary professionals and dog trainers/behaviourists can leverage increased functional walking for the needs of the dog. Dog trainers and behaviourists also have a strong role to play in working with owners of dogs with behavioural/training difficulties so that they can have a more relaxing and recreational walking experience. Health professionals, in particular mental health, can encourage and educate regarding the recreational benefits to the owner. Perhaps the most important roles though are that of policy-makers and municipal planners in encouraging dog walking through appropriate environmental design in order to enable a recreational experience.

Our study highlights the importance of the physical environments in which dog walking occurs. The design and planning of these environments are likely to affect the frequency of dog walk that are undertaken (Westgarth *et al.*, 2014; Christian *et al.*, 2018). Our study has shown that dog owners want to walk in environments that facilitate positive emotions for both themselves and their dogs. This means being aesthetically pleasing and interesting, safe, and including provision for off-leash running and exploring. As previously highlighted (Fletcher and Platt, 2018), on-leash street walks are considered boring for the dog and are not highly motivating for the owner, and our findings add that these walks are classed as merely functional at least in the UK (it remains to be investigated if the same applies in countries where off-leash walking is less normalized) The provision of locations considered suitable for dog walking is so important that owners who have a choice will drive to a more suitable location, or pick areas to live considered to have good dog walking. Dog walking sites also need to be physically accessible for easy use by those with health conditions or walking with small children; this requires for them to be designed as ‘spaces for all’, with accessible flat paths, and spaces for children and dogs to share rather than necessarily segregating them apart from each other. Confining off-leash facilities to small enclosed dog parks were deemed not suitable for either dogs or owners; this could explain why provision of off-leash parks (or areas of parks) have not been shown to result in clear increases in dog walking (McCormack *et al.*, 2011, 2016). When provision of an ideal environment for dog walking is not possible, perhaps at least the provision of dog-friendly amenities and destinations to visit may enrich the recreational nature of an otherwise functional walk.

This study has a number of strengths. The data were based upon in-depth interviews with a relatively large sample of people and combined with observational data. This ethnographic approach enabled interactions between household members to be observed including discussions between family members about the dog’s walk. Caring for a dog involves negotiation between family members and each family member had their own perspective on what it is like to own a dog. The participants included both male and female adults and children, across different social classes, and both people who rarely walked with the dog as well as those who were regular dog walkers. In many cases, an actual dog walk was experienced with them, as opposed to just talking about it theoretically, providing a richer study of the topic (Campbell *et al.*, 2016). However, this study has limitations. As with all studies, the decision to participate in the study rests with the individual, making the sample self-selected. Given that dog ownership encompasses individuals at all stages of the life course, all ethnicities and all social circumstances, more detailed focused studies are required to examine how this division between functional and recreational walks might extend to other groups of dog owners.

In conclusion, functional dog walks are performed for the sake of the dog and human walking activity is incidental. Recreational walks meet the needs of both dogs and humans and are performed as an integral practice of walking together through an environment suited to the needs and outcomes of this activity. Functional and recreational dog walks may be performed at different times both within and between individuals, and in different physical environments. These two types of dog walking have implications for the design of interventions and environments aiming to promote dog walking. Levering benefits to the dog from walking is likely to encourage initiation of owner dog walking activities at a smaller, functional and scale. The provision of physically accessible, safe, aesthetically pleasing and dog-appropriate (off-leash) physical environments for dog owners to enjoy walking in, are likely to be the most encouraging for maintaining long-term physical activity promotion.

## ETHICAL APPROVAL

The study was approved by University of Liverpool Veterinary Ethics Committee (Project code VREC121).

## FUNDING

This research was funded by a Medical Research Council Population Health Scientist Fellowship (Grant ref: G1002402) held by the primary author.

## CONFLICT OF INTEREST STATEMENT

C.W. and R.C. have received research grant funding from pet food companies. R.C. now works for Dogs Trust but did not do during this study.
